# The solar magnetic activity band interaction and instabilities that shape quasi-periodic variability

**DOI:** 10.1038/ncomms7491

**Published:** 2015-04-07

**Authors:** Scott W. McIntosh, Robert J. Leamon, Larisza D. Krista, Alan M. Title, Hugh S. Hudson, Pete Riley, Jerald W. Harder, Greg Kopp, Martin Snow, Thomas N. Woods, Justin C. Kasper, Michael L. Stevens, Roger K. Ulrich

**Affiliations:** 1 High Altitude Observatory, National Center for Atmospheric Research, PO Box 3000, Boulder, Colorado 80307, USA; 2 Department of Physics, Montana State University, Bozeman, Montana 59717, USA; 3 Cooperative Institute for Research in Environmental Sciences, University of Colorado, Boulder, Colorado 80205, USA; 4 Lockheed Martin Advanced Technology Center, 3251 Hanover Street, Building 252, Palo Alto, Colorado 94304, USA; 5 Space Sciences Laboratory, University of California, Berkeley, California 94720, USA; 6 Predictive Science Inc., 9990 Mesa Rim Road, Suite 170, San Diego, California 92121, USA; 7 Laboratory for Atmospheric and Space Physics, University of Colorado, 1234 Innovation Drive, Boulder, Colorado 80303, USA; 8 Harvard-Smithsonian Center for Astrophysics, 60 Garden Street, Cambridge, Massachusetts 02138, USA; 9 Department of Atmospheric, Oceanic and Space Sciences, University of Michigan, Ann Arbor, Michigan 48109, USA; 10 Division of Astronomy and Astrophysics, University of California, Los Angeles, Colorado 90095, USA

## Abstract

Solar magnetism displays a host of variational timescales of which the enigmatic 11-year sunspot cycle is most prominent. Recent work has demonstrated that the sunspot cycle can be explained in terms of the intra- and extra-hemispheric interaction between the overlapping activity bands of the 22-year magnetic polarity cycle. Those activity bands appear to be driven by the rotation of the Sun's deep interior. Here we deduce that activity band interaction can qualitatively explain the ‘Gnevyshev Gap'—a well-established feature of flare and sunspot occurrence. Strong quasi-annual variability in the number of flares, coronal mass ejections, the radiative and particulate environment of the heliosphere is also observed. We infer that this secondary variability is driven by surges of magnetism from the activity bands. Understanding the formation, interaction and instability of these activity bands will considerably improve forecast capability in space weather and solar activity over a range of timescales.

## Abstract (web summary)

The origins of the Sun's periodic activity, such as sunspot cycles, are poorly understood. McIntosh *et al.* posit that the rotational forcing of the activity bands comprising the 22-year magnetic cycle undergoes shorter-term variations, driving magnetic flux surges that impact solar output on those timescales.

## Graphical abstract

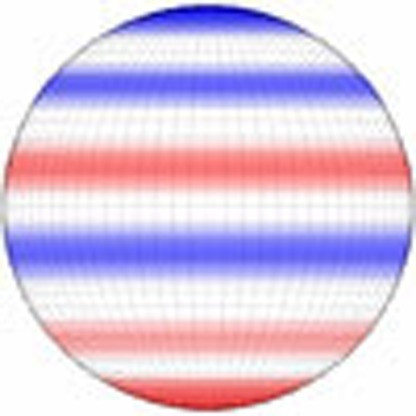

The obvious hemispheric asymmetry of the solar atmosphere over the past several years (2009–2014) has generated a significant amount of interest in the heliophysics community^1^. Indeed, the asymmetric magnetic evolution of the Sun's northern and southern hemispheres enabled the recent demonstration that the 22-year magnetic polarity cycle strongly influences the occurrence, and distribution of the sunspots which form the 11(-ish)-year solar activity cycle^2^—an observational result that challenges the current understanding of the Sun's magnetism factory, the solar dynamo^3^.

McIntosh *et al.*^2^ illustrated that the twisted toroidal bands of the 22-year magnetic polarity cycle are embedded in the Sun's convective interior and first appear at high latitudes (∼55°) before travelling equatorward. These bands interact with the oppositely polarized magnetic band from the previous cycle at lower latitudes in each hemisphere. The interaction of these activity bands is illustrated in Fig. 1 and modulates the occurrence of sunspots on the low-latitude bands (which have opposite magnetic polarity and sense of handedness) until they eventually cancel across the equator (as occurs in 1998). This equatorial cancellation signals the end of the sunspot cycle and leaves only the higher-latitude band in each hemisphere. Sunspots rapidly appear and grow on that band for several years until a new oppositely signed band appears at high latitude (for example, 2001 in the north, and 2003 in the south)—an occurrence that defines the maximum activity level of that new cycle and triggers a downturn in sunspot production. The perpetual interaction of these temporally offset 22-year activity bands drives the (quasi-)11-year cycle of sunspots that form the decadal envelope of solar activity. The observational evidence presented by McIntosh *et al.*^2^ points to the rotational energy at the bottom of our Star's convection zone as being the major driver of the Sun's long-term evolution.

Rotating atmospheres, like that of the Earth and the giant planets of our solar system, often exhibit shorter-timescale global-scale phenomena such as Kelvin and Rossby waves^4^,^5^, which are important for the transport and regulation of energetics in those systems^6^. In the following analysis we argue, based on a host of observations displaying (quasi-)periodicities of significantly shorter—but commensurate amplitude—to the well-established decadal-scale ‘solar cycle' variability, that the Sun is no different. It is possible that the convecting, magnetized, ‘ocean' beneath the Sun's optical surface could exhibit similar global-scale wave behaviour to those readily observed in our atmosphere and other planetary atmospheres in the solar system^7^,^8^. Such phenomena could drive marked changes in the Sun's interior and the rate at which magnetic flux pierces our star's photosphere. Once forced into the outer solar atmosphere, that magnetic flux will strongly affect the radiative, particulate and eruptive output of the Sun.

## Results

### Activity band interaction and the Gnevyshev Gap

**Figure 2:**
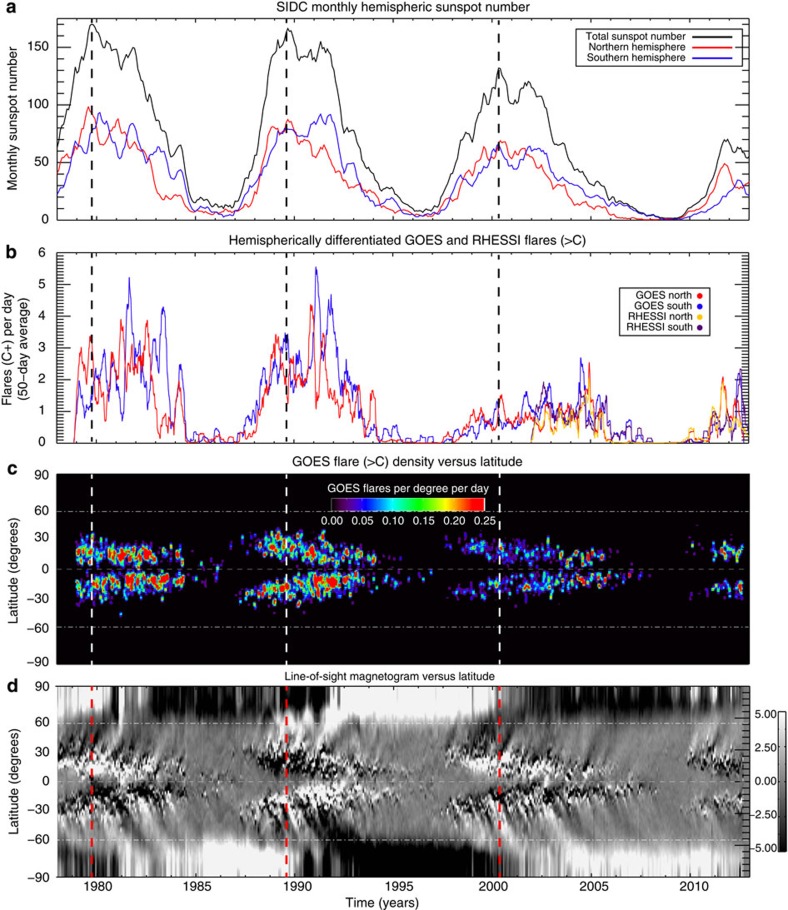
Magnetic variability over the last three decades. Comparison of the variation in (monthly) sunspot number (SSN) and flare record with the ‘butterfly' diagram of the photospheric magnetic field over the past three solar cycles. (**a**) Total (black) and hemispheric (red—north; blue—south) monthly sunspot numbers (hSSN) from the Solar influences data center (SIDC). (**b**) Variation of the hemispheric daily rate of flares larger than ‘B' magnitude in the GOES (red—north; blue—south) and RHESSI (orange—north; purple—south) records. Note the strong modulation in the flare rate, the hemispheric differences in flare rates and that flare maximum does not occur at the same time as sunspot maximum—over the record shown, the flare activity maximum occurs several years post sunspot maximum. (**c**) Latitude–time distribution of the GOES flares of **b**. (**d**) Latitude–time variation of the photospheric magnetic field at the central meridian. Note the strong correspondence between the poleward pulses of photospheric magnetism and the surges in flare activity from **c** and **b**. All panels show a thick vertical dashed line indicating the time of sunspot maximum and the lower two panels show dot-dashed lines at 55° to delineate high- and low-latitude variation.

Variations of significantly shorter period than the canonical (11-year) envelope of solar variability are visible in the Sun's flaring activity (Fig. 2). The figure paints a canvas of the Sun's magnetism over the past 35 years—the last three-plus sunspot cycles. The dwindling number of sunspots and flares occurring on the Sun over that period is clearly indicating a net downturn in solar activity. We see that the peak flare rate occurs at a different time from the sunspot maximum—often a few years later—an observational phenomenon known as the ‘Gnevyshev Gap'^9^,^10^. Superimposed on that decadal-scale envelope we see that flares and (the monthly number of) sunspots quasi-periodically surge in number. These well-documented^11^,^12^ surges in solar activity, resulting from a 10–15% increase in sunspot numbers, result in a doubling or tripling of the flare rate over the course of several months. In addition, a latitude–time probability density function of flare activity from the National Oceanic and Atmospheric Administration (NOAA) record (Fig. 2c), indicates that the spatio-temporal clustering of strong flares shares a common origin with the ‘herringbone' pattern seen in magnetic butterfly diagrams (Fig. 2d). That herringbone pattern appears to propagate from mid-to-high latitudes on a quasi-periodic basis from a common point of origin with the flare clusters. The relationship between the flare clusters and the root of the herringbone pattern is due to their association with sunspots. The correspondence of the two implies that the magnetic fields at the root of the system (in the magnetic activity bands) are being perturbed in a quasi-periodic manner by some physical process related to the evolution of the activity bands themselves. That process produces such a rapid and strong increase of magnetic flux emergence that is hard to reconcile with any known convective or shear phenomena occurring in the surface, or near-surface, layers.

### Short-term variability in cycle 23

**Figure 3:**
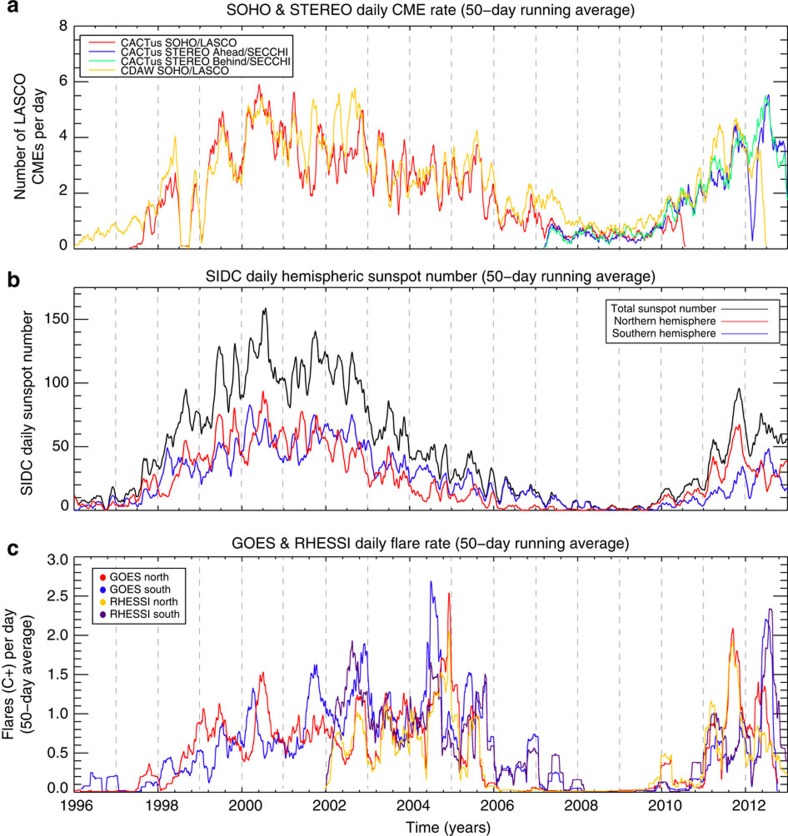
Variability of the Sun's eruptive output over solar cycle 23. Comparison of the variation in the CME and flare rates over solar cycle 23 with the modulation in the (daily) sunspot number. (**a**) Variation in the (whole Sun) daily CME rates as detected by the CACTus^44^ and CDAW^13^ methods for the SOHO (red—CACTus; orange—CDAW) and the twin STEREO (blue—‘ahead'; green—‘behind') coronagraphic data sets. (**b**) SIDC- Solar influences data center. Total (black) and hemispheric (red—north; blue—south) daily sunspot numbers—compare with the monthly counterpart in Fig. 2. (**c**) Variation of the hemispheric daily rate of flares larger than ‘B' magnitude in the GOES (red—north; blue—south) and RHESSI (orange—north; purple—south) records. As in Fig. 2, there is considerable lag between (total) sunspot maximum with the CME and flare series—occurring late in the descending phase. Almost every bump and wiggle in the sunspot number shows a corresponding surge in CME and flare activity—these surges can be as large amplitude as a doubling of the sunspot number or flare/CME rate over the course of only a few months before recovering. The panels of the figure show a set of dashed fine vertical lines that are 12 months apart and act as a timescale reference. Each timeseries shown in these panels is a 50-day running average over the original. The CME timeseries are not separated by hemisphere due to the uncertainty in determining the actual CME location from only plane-of-the-sky coronagraphic observations.

Studying the last solar cycle in more detail, Fig. 3 compares the daily coronal mass ejection (CME) rates inferred from the National Aeronautics and Space Administration (NASA) SOHO and STEREO spacecraft, the sunspot number and the flare rates determined from the GOES archive (Fig. 2) and the NASA RHESSI spacecraft. We see that two different CME detection algorithms^13^,^14^ applied to the SOHO data set arrive at very similar whole-Sun statistics. Those also match the CME statistics derived from STEREO observations from late 2006 to the present^13^. An important detail to note here is that the STEREO spacecraft spent almost their entire mission time off of the Sun–Earth line, strengthening the perception that the phenomena driving the changes in CME rates are global in nature—being independent of the observer's specific (heliocentric) longitude. Due to uncertainties in identifying the absolute origin of CMEs on the solar disk, especially those from the far side (which nevertheless are detected by white-light coronagraphs), we do not attempt to identify the events from the northern and southern hemisphere. As such, the CME statistics reflect the behaviour of the ‘whole Sun'.

We see that the peaks in the total sunspot number have corresponding peaks in the CME rate. The surges in the daily sunspot number can be as large as 30% and they can lead to a 100% increase in the daily CME rate. The same strong correspondence is visible for the flare rate. The relationship between the disk-integrated CME rate and the hemispheric rates of sunspot and flare formation highlight a critical property in disk-integrated quantities—they will typically exhibit shorter period variations than hemispherically resolved ones. The phase offset between the two hemispheres will determine the resulting ‘hybrid' period observed. In this case, we see that marked increases in surface magnetism lead to a profound increase in the rate of eruptive phenomena.

**Figure 4:**
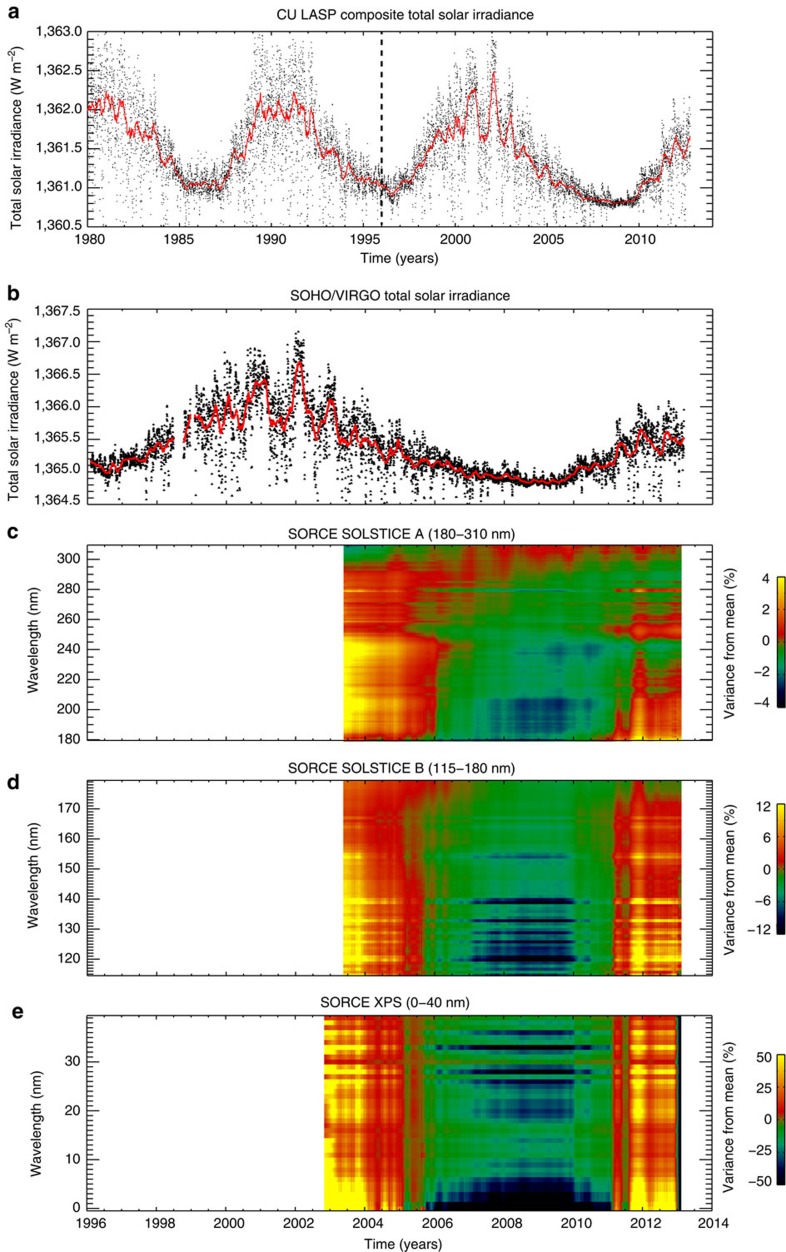
Variability in the total solar irradiance over the past three decades in comparison with the variance in components of the solar spectral irradiance over solar cycle 23. (**a**) The University of Colorado TSI composite^10^ in comparison with (**b**) the SOHO/VIRGO TSI over solar cycle 23—the thick vertical dashed line marks the start of the SOHO*/*VIRGO record used. In both cases, the thick red lines are the 50-day running average over the measurements. While the mean solar minimum to solar maximum change in TSI is ∼1 Wm^−2^, there is a shorter-period modulation variation visible in the TSI over the entire time frame. That variation, of the same magnitude as the decadal variation, is better defined in solar cycle 23 due to refinement in instrument design and calibration^10^. (**c**–**e**) Percentage variation in different bands (relative to the mean spectrum) of the solar spectral irradiance from the SORCE spacecraft from the far-ultraviolet, ultraviolet SOLSTICE measurements. As we move to shorter wavelengths, the degree of variation in one of the surges in solar radiation increases from a few to 50%. XPS, X-ray photoelectron spectroscopy.

It is not only eruptive phenomena that exhibit variations of similar magnitudes and timescales. Figure 4 shows the (disk-integrated) total solar irradiance (TSI) and components of the spectral solar irradiance measured from space. The variance in the TSI is visible over the entire record (Fig. 4a), but as the measurement has been refined and systematic errors in it have been reduced (especially with the addition of SOHO/VIRGO to the record)^15^, we see that the amplitude of the short-term variability is ∼1 Wm^−2^—equivalent to the variation over the whole solar cycle (Fig. 4b). We see that the ultraviolet (Fig. 4c), extreme ultraviolet (Fig. 4d) and X-ray (Fig. 4e) components of the spectral solar irradiance (as measured by the SORCE spacecraft) show variability over the mean spectrum from a few to almost 100% during the activity surges.

### Variability in the solar wind and fast wind source regions

**Figure 5:**
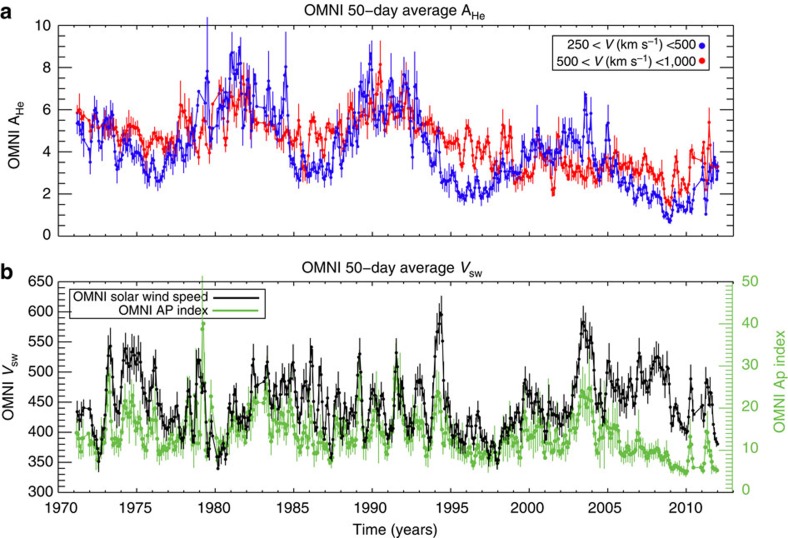
Variation of solar wind and geomagnetic properties over the past four decades. The data presented are from the NASA/GSFC Space Physics Data Facility OMNI database (http://omniweb.gsfc.nasa.gov/). (**a**) Variation in 50-day running averages of the fast (red) and slow (blue) solar wind helium abundance (A_He_; ref. 17)—a proxy of plasma heating at the base of the solar wind^16^. (**b**) Variation in the 50-day running averages of the solar wind speed (*V*_sw_; black) and geomagnetic storm Ap index (green). Note the steady drop in A_He_ over the time frame and the strongly correlated quasi-periodicities in all four quantities where the surges in *V*_sw_, A_He_ and Ap are of the order 100 km s^−1^, 15 and 50%, respectively. The error bars in the plot reflect the variance of the signal over the 50-day running window.

Similarly, Fig. 5 shows another highly modulated facet of quiescent solar behaviour that illustrate these global surges in magnetism—properties of the solar wind and its geomagnetic impact. The abundance of helium is a marker of magnetic activity in the solar atmosphere^16^,^17^. While the amount of helium in the fast and slow solar wind shows a strong decline over the past three decades^1^; (cf. Fig. 2b) we can also see the clear 20–50% swings of short-term variability. Short-term variability is also visible in the speed of the solar wind and the Ap geomagnetic activity index that it influences (Fig. 5b)—noting that solar wind characteristics are strongly impacted by the three-dimensional geometry of the heliosphere's magnetic field, and where the spacecraft sampling interplanetary space are situated. The coherent variation of the solar wind speed^18^ indicates that the processes governing the shape of the magnetosphere^19^ and heliosphere are being driven by the surges in magnetic variability.

**Figure 6:**
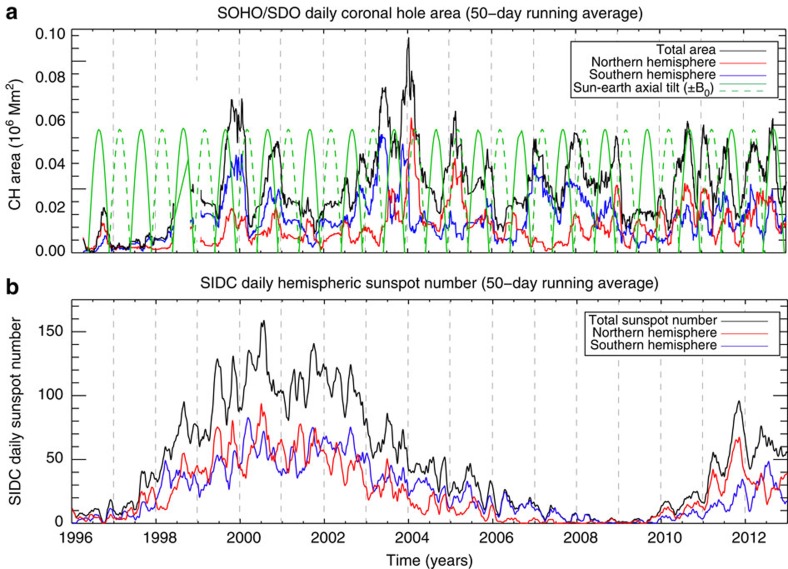
Variation in the low-latitude coronal hole area and the sunspot number over solar cycle 23. (**a**) Using the CHARM^21^ automated SOHO/EIT and SDO/AIA coronal hole-detection algorithm, we show the variation of the 50-day running average in the total (black), northern (red) and southern (blue) hemispheric coronal hole areas below 55° latitude. For reference, the seasonal variation in the Sun's axial tilt relative to the Sun–Earth line is shown (green—the dashed green line shows amplitude of the negative tilt). (**b**) Solar influences data center (SIDC). Total (black) and hemispheric (red—north; blue—south) daily sunspot numbers as in Fig. 3. Like the flare and CME timeseries shown above, the coronal hole areas peak after solar sunspot maximum (∼2004). A comparison of the hemispheric sunspot and coronal hole areas shows a systematic lag in the peaks of the latter (∼6 months). The 2004 peak in coronal hole area corresponds to the peaks in the Ap index and solar wind speed of Fig. 5. The panels of the figure show a set of dashed fine vertical lines that are 12 months apart and act as a timescale reference.

These periodic changes in the morphology of the coronal (and heliospheric) magnetic field can be inferred from Fig. 6 where we contrast the variation of total and hemispheric areas of low-latitude (<55°) coronal hole areas with hemispheric sunspot numbers (cf Fig. 3) and the solar *B*_0_-angle (the heliographic latitude of the central point of the solar disk). The seasonal variation of the latter modulates the visible area of the solar disk^20^, but cannot exclusively explain the strong periodicities in the hemispheric coronal hole areas or their time-varying phase relationship. Further, the hemispheric coronal hole areas appear (on-average) to lag the sunspot numbers by a few months—the former are typically a higher-latitude phenomena than the sunspot band^2^. This strengthens the premise that at least some of the magnetic flux which forms coronal holes is the result of active region flux diffusion^21^. The increase in coronal hole area during the declining phase of the sunspot cycle (with noticeable peaks in 2003–2004) is another well-observed phenomenon^22^ but is more related to the gross interaction of the 22-year activity bands that we have discussed earlier.

### Quasi-periodic variability of solar magnetism

**Figure 7:**
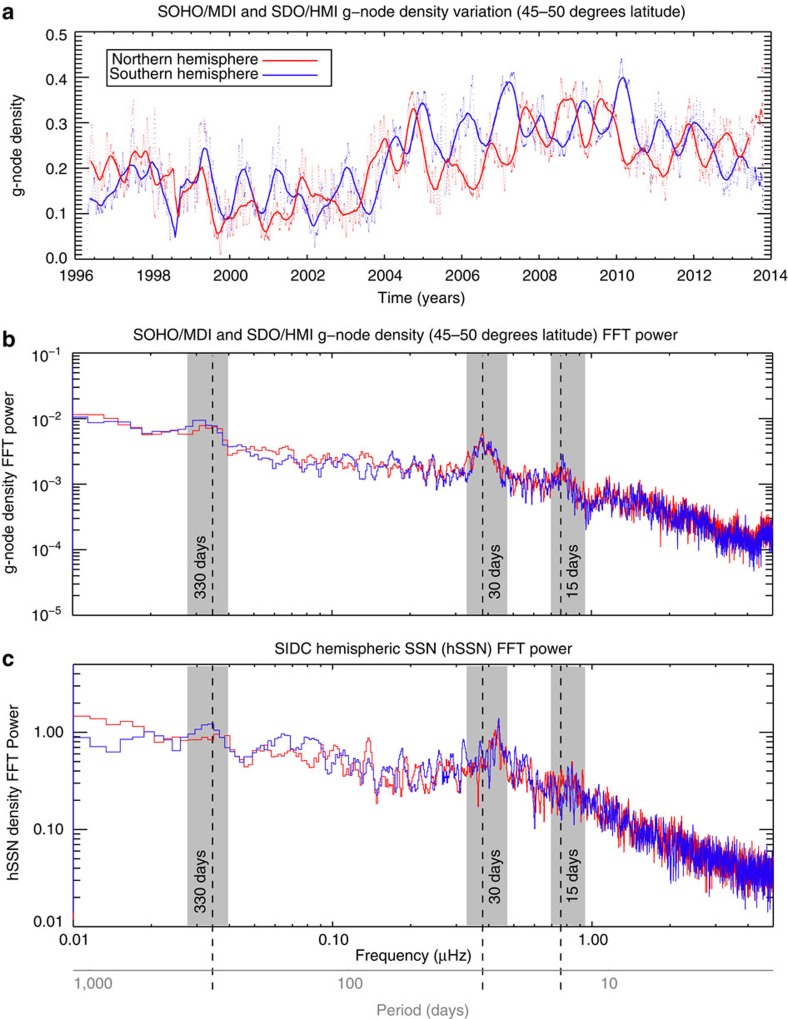
Variation in the deep-rooted small-scale solar magnetic features over solar cycle 23. (**a**) Variation in the density of giant convective cell vertices (g-nodes) averaged over 45–50° latitude in the northern (red) and southern (blue) hemispheres from the SOHO Michelson Doppler Imager and SDO Helioseismic Magnetic Imager—markers of deep-rooted solar magnetism that belong to the toroidal magnetic flux systems of the 22-year magnetic activity cycle^2^. The small dots are individual daily averages, while the thick lines are the corresponding 50-day running average. As in Fig. 6, the variable phase of the timeseries in each hemisphere is strongly indicative of a solar origin for these phenomena and not some orbital or Sun–spacecraft distance variability. The periods where the hemispheres vary in phase correspond to the times of strongest modulation in the energetic parameters shown in the figures above. (**b**,**c**) Fast Fourier transform (FFT) power spectra of the northern and southern hemispheric g-node timeseries, when compared with counterparts for the daily hemispheric sunspot number, respectively, (Fig. 3) show broad peaks of significant power occurring throughout the timeseries, especially those centred on 330 days, 30 days and 15 days in the shaded regions.

Magnetic fields on smaller spatial scales than coronal holes and sunspots display similar periodicities to their larger brethren throughout the solar cycle. Figure 7a shows the evolution in number density of the magnetic elements associated with the vertices of the giant convective scale^2^,^23^. This convective scale is driven by the rotation of the deep radiative interior, and these ‘g-nodes'^23^ are believed to be anchored close to the bottom of the convection zone's boundary with the radiative interior. The number of g-nodes in each hemisphere waxes and wanes over the course of solar cycle 23, in addition to being strongly variable over shorter timescales. g-Node densities also display a varying phase offset between the two solar hemispheres. The Fourier power spectra (Fig. 7b) of the hemispheric g-node density and (daily) sunspot timeseries have very similar characteristic timescales as indicated by the grey-shaded regions in the figure. The short-period (higher frequency) envelope peak of 11–16 days is approximately one half of the rotational period (24–35 days). This indicates that magnetic patterns do not diffuse immediately on the Sun's surface. The slight offset between peaks in the low- (28 days) and high-latitude (30 days) period is consistent with observed solar differential rotation^24^. The broad peak centred around 330 days is common to the timeseries, although the southern hemisphere seems to be shifted further and is consistent with the analysis of Getko^25^,^26^. This appears to be the primary (quasi-)periodicity of the magnetic surges that shape the heliosphere and drive the host of energetic phenomena observed as described above. Wavelet analyses of these timeseries (see the Methods section; Supplementary Figs 1–3) demonstrate that the aforementioned peaks occur with a 99% confidence level.

## Discussion

The physical origin of these strong quasi-periodic surges in the Sun's magnetism is not known. However, their effect on the outer solar atmosphere and on the geospace environment is profound. Their existence has been documented extensively since the start of the space-age. For example, strong quasi-periodicities that are longer than the Sun's rotation rate have been amply documented in the literature for sunspot areas^27^, flares^11^, CMEs^28^ and major geomagnetic storms^29^,^30^, but it is likely that any property of the outer solar atmosphere that is dependent on magnetism will show a response of varying degree^1^ and that extends to the interplanetary magnetic field^31^.

**Figure 8:**
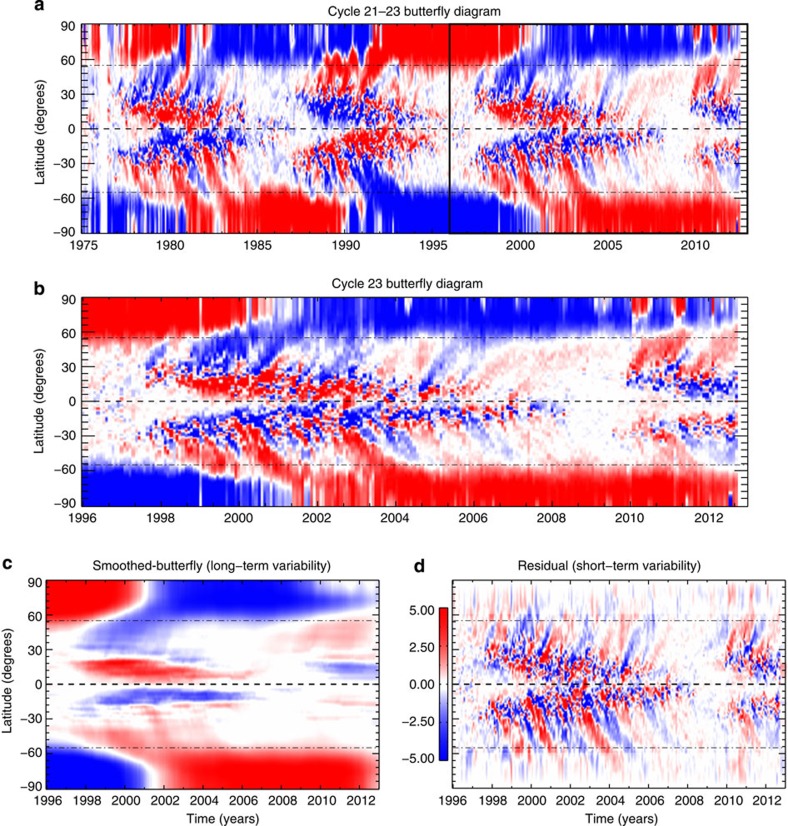
Gross decomposition of the surface magnetism of the past four decades into short-term and long-term variability components. The decomposition follows the method of Ulrich^30^. (**a**) The pattern of photospheric magnetic field in a latitude–time plot constructed using Carrington rotation (28-day) sampling of the central meridian field. The inset region shown as a black rectangle outlines the latitude–time plot shown in **b**. (**c**) Hundred-day average field from **b** and the residual between that 100-day average and the original latitudinal variation (**d**). The average and residual correspondingly decompose the surface magnetism into the space climate and space weather modulations that bathe the earth in radiation, particles and disruptive events. The poleward surges of magnetism shown in **d** are directly related to the strong modulation shown in the figures above. For illustration, the equator and 55° lines are shown as black dashed and dot-dashed lines, respectively.

As we have noted above, it is unlikely that a strong modulation in the number of sunspots can be easily explained by processes in the near-surface layers of the Sun. However, considering a spatio-temporal decomposition of solar surface magnetism^32^ can provide some interpretative guidance. Figure 8 shows Ulrich's decomposition of the photospheric butterfly diagram (Fig. 2d) into a long-term smoothed radial field and a residual. The latter reveals poleward-propagating features in each hemisphere. The primary signal in the (smoothed) butterfly diagram is divided into high- and low-latitude evolution at ∼55° latitude^2^, both alternate in sign and are long lived—the lower-latitude pattern propagates equatorially. This pattern is associated with the interacting activity bands of the 22-year magnetic polarity cycle described by McIntosh *et al.*^2^ The secondary pattern, visible in the residual between the primary pattern and original data set, is poleward propagating, is not symmetric across the equator and has a much shorter timescale than the former. Ulrich^32^ notes that the latter pattern is not compatible with simple (single meridional cell) surface advection of magnetic flux.

We infer that the interaction of the oppositely signed, long-lived activity bands in each hemisphere as discussed by McIntosh *et al.*^2^ can help explain why the flare, CME and coronal hole timeseries peak so long after (total) sunspot maximum. The latitudinal interaction—via flux emergence—of the activity bands in each hemisphere must peak at some point after the time it starts propagating equatorward, the time that defines solar maximum^2^. Such an interaction of the activity bands, combined with the phase difference of hemispheric evolution^1^, can explain Gnevyshev's observational findings^10^ where hemispheric asymmetry alone cannot^33^. Substantial numerical simulations of the interaction between deep-rooted magnetic flux and convection^34^ are positive initial steps in exploring the range of variability in decadal-scale solar output by placing magnetic flux systems in a rotating convective envelope.

In addition to the decadal envelope of solar activity, there is a clear, strong, variability of the magnetic flux in each solar hemisphere of approximately 1 (terrestrial) year. We propose that the process at the root of the short-term propagating pattern shown in Fig. 8 is responsible for the surges in solar activity and the latitudinal variation in the proxies that we have noted above.

Figure 7 permits a phenomenological explanation of the quasi-periodicities (of order 150 days) that have been observed in a large number of heliospheric quantities by Rieger and others^9^,^10^,^11^,^25^,^26^,^27^,^28^,^29^,^35^. Those are ‘hybrid' periodicities—a consequence of the phase relationship between the short-term variability in each solar hemisphere. In short, the longer-period hemispheric timeseries from each hemisphere will combine to produce a shorter period (higher frequency) whole-sun timeseries—consider our earlier example for flares and CMEs. Indeed, the same principle can possibly explain the quasi-periodicities seen in helioseismic measurements of the deep convection zone^36^—if our assertion that the phenomena at the root of this problem occur on the activity bands, near the base of the convection zone, is correct. In this case, noting that (standard) global helioseismology analyses impose hemispheric symmetry, the phase of the timeseries in each hemisphere is critical. Only in the earlier part of cycle 23 (1998–2002) would the two hemispheres constructively create a signal that can be detected using this method, as the hemispheres were then approximately in phase.

So, what are the poleward-propagating excursions seen in Fig. 8 and how are they driven? The simplest possible explanation is one where the surges in solar magnetism periodically load more flux into the Sun's surface layers. Once those magnetic regions begin to decay and diffuse over time^37^, the surface meridional circulation^21^ is loaded with magnetic flux that is then carried poleward. While this appears straightforward, it does not answer the second and most important part of the question—what drives the surges of magnetism?

One possible explanation follows from the deliberations of Howe *et al.*^36^ Howe *et al.* indicate that there are global-scale waves and instabilities that propagate in the shear layer known as the tachocline^3^ at the bottom of the convection zone—where the activity bands appear to be rooted. We then observe the impact of those waves and instabilities on the surface magnetism of our star through their modulation of the global magnetic flux emergence process^38^,^39^.

What could these perturbations to the magneto-convective system be? The Earth's mantle, ocean and thermosphere/stratosphere exhibit global-scale waves that are driven by the rotation of the planet at shear interfaces, or Rossby waves^40^,^41^, like the tachocline. The action of such energetic interface waves in the solar interior could dynamically modify the buoyancy characteristics of the flux tubes present in the region above^39^. Theoretical efforts indicate that magnetized Rossby waves with periods of order several hundred days are highly likely^42^,^43^ in a non-zero thickness tachocline^38^. Whether or not the surges of magnetism are caused by large-scale Rossby-like waves in the Sun's convective interior, we have seen that they force large upswings in solar activity of quiescent and explosive nature. The period of the surges in each solar hemisphere is close to 1 terrestrial year, and the hemispheric phase relationship influences the period of the disturbances felt in the heliosphere. Significant research remains to be done to determine whether the apparent periodicity is a fundamental characteristic of our star's deep interior, and to understand the processes responsible for producing it.

To summarize, we have inferred that the interaction of the activity bands belonging to the Sun's relentless 22-year magnetic polarity cycle shape the decadal-scale variability of solar activity^1^,^2^. In addition, there is a quasi-annual modulation of solar activity—with a magnitude commensurate to that of the decadal variability—which appears to be driven by surges of magnetic flux originating in those activity bands.

The growing dependence of our civilization on technology susceptible to space weather should motivate investigations into the rotational forcing of the Sun's deep convection zone by the radiative zone. Specifically, challenging simulations of activity band formation, intra- and extra-hemispheric activity band interaction and the zoo of rotational-gravity-buoyancy waves that interact with those activity bands are required. These factors appear to be key drivers of solar variability on decadal and annual timescales. A better understanding of the processes responsible for modulating the decadal variability and the (quasi-)annual ‘seasons' of solar activity will yield a significantly increased forecast skill for solar activity in parallel with continued observational monitoring.

## Methods

### Periodicities

Figure 7 presents a Fourier analysis of the hemispheric g-node and sunspot variability. The figure indicates the prevalence of a ∼330-day quasi-periodicity in those timeseries. In the following discussion we employ wavelet transform techniques, as presented by Torrence and Compo^43^, as a means to demonstrate the significance of the timescales in the hemispheric g-node (Supplementary Fig. 1), daily hemispheric sunspot number (Supplementary Fig. 2) and the SOHO/VIRGO TSI measurements (Supplementary Fig. 3) that appear in Fig. 4b.

Using the Morlet wavelet as a model representation of the oscillatory signals observed, we construct the wavelet power spectra of each timeseries. In the most general sense a wavelet power spectrum can be thought of as an image that indicates the strength and the duration that an oscillation of a particular period is present in the timeseries. It is a particularly powerful tool for the analysis of timeseries that exhibit mixed periods and quasi-periodicities as is often the case in many solar phenomena.

For the wavelet power spectra shown in Supplementary Figs 1–3, we can clearly see that periodicities of order 330 days are present at an 99% confidence level. The 99% confidence level in the wavelet power spectra shown is indicated by a solid, thick, closed contour. Statistical confidence in this case is computed with respect to a red noise model of the spectral background—a spectral background that has increasing power with decreasing frequency. This model is common for most solar and geophysical data sets^43^ and is adequate for the present application. It appears that the ∼30- and ∼15-day quasi-periodicities also have strong wavelet power in the timeseries studied, although the wavelet power does not always meet the 95% confidence criteria. This can be most easily seen in Supplementary Fig. 2 for the hemispheric daily sunspot number. However, the signal and wavelet power spectra are likely impacted at shorter periods by our earlier choice to study a 50-day running average of the sunspot timeseries.

The cross-hatched areas in the wavelet power spectrum define the ‘cone of influence.' The interpretation of the cone of influence is relatively straightforward—the signal in the cross-hatched area may not be entirely reliable because of the influence of edge effects of the timeseries. Because these are finite-length timeseries, errors will occur at the beginning and end of the wavelet power spectrum, as the Fourier transform used in the Wavelet method assumes that the data are cyclic^43^. The solution used in these particular wavelet methods pads the end of the timeseries with zeroes before performing the wavelet transform and removes them afterwards. In the examples shown, the timeseries are padded with sufficient zeroes to bring the total length *N* up to the next-higher power of two—this limits any edge effects and speeds up the Fourier transform at the core of the computation.

### Data sources

The data used in this paper are openly available from the NGDC, SOHO, SDO and the Virtual Solar Observatory (http://virtualsolar.org) data archives.

## Author contributions

S.W.M. performed data analysis, figure construction and wrote the manuscript. R.J.L. assisted S.W.M. with writing, data analysis and discussion. L.D.K. developed the coronal hole-detection algorithm and related statistics. A.M.T., H.S.H. and P.R. made significant contributions to the structure and discussion presented in the manuscript. J.W.H., G.K., M.S. and T.N.W. developed and assisted in the construction of the TSI/SSI timeseries presented. J.C.K. and M.L.S. performed the original analysis shown in Fig. 5, and R.K.U. assisted in the construction of Fig. 8.

## Additional information

**How to cite this article:** McIntosh, S. W. *et al.* The solar magnetic activity band interaction and instabilities that shape quasi-periodic variability. *Nat. Commun.* 6:6491 doi: 10.1038/ncomms7491 (2015).

## Supplementary Material

Supplementary Information Supplementary Figures 1-3

## Figures and Tables

**Figure 1:**
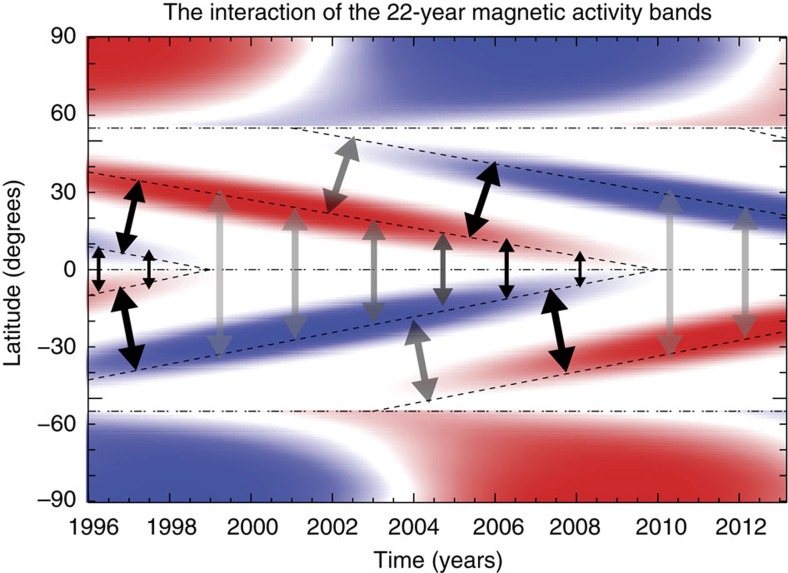
Activity band interaction. A data-derived schematic of the the latitudinally interacting activity bands of the 22-year magnetic polarity cycle, as introduced by McIntosh *et al.*^2^ The bands, visualized here in the radial component of the magnetic field, of opposite polarity start their migration towards the equator from high latitudes in each hemisphere and take ∼19 years to reach their termination. The arrows illustrate some of the possible interactions between the bands within, and without, their hemisphere while the opacity of the arrows indicate the (potential) strength of the interaction between the two. This figure is adapted from Fig. 8 of ref. 2. Copyright 2014 by The American Astronomical Society.
