# Supplementary material for: The solar magnetic activity band interaction and instabilities that shape quasi-periodic variability
Source: Nat Commun. 2015 Apr 7;6:6491. doi: 10.1038/ncomms7491 (PMC4396379; doi:10.1038/ncomms7491)
Supplement: Supplementary Information — Supplementary Figures 1-3 [file ncomms7491-s1.pdf]

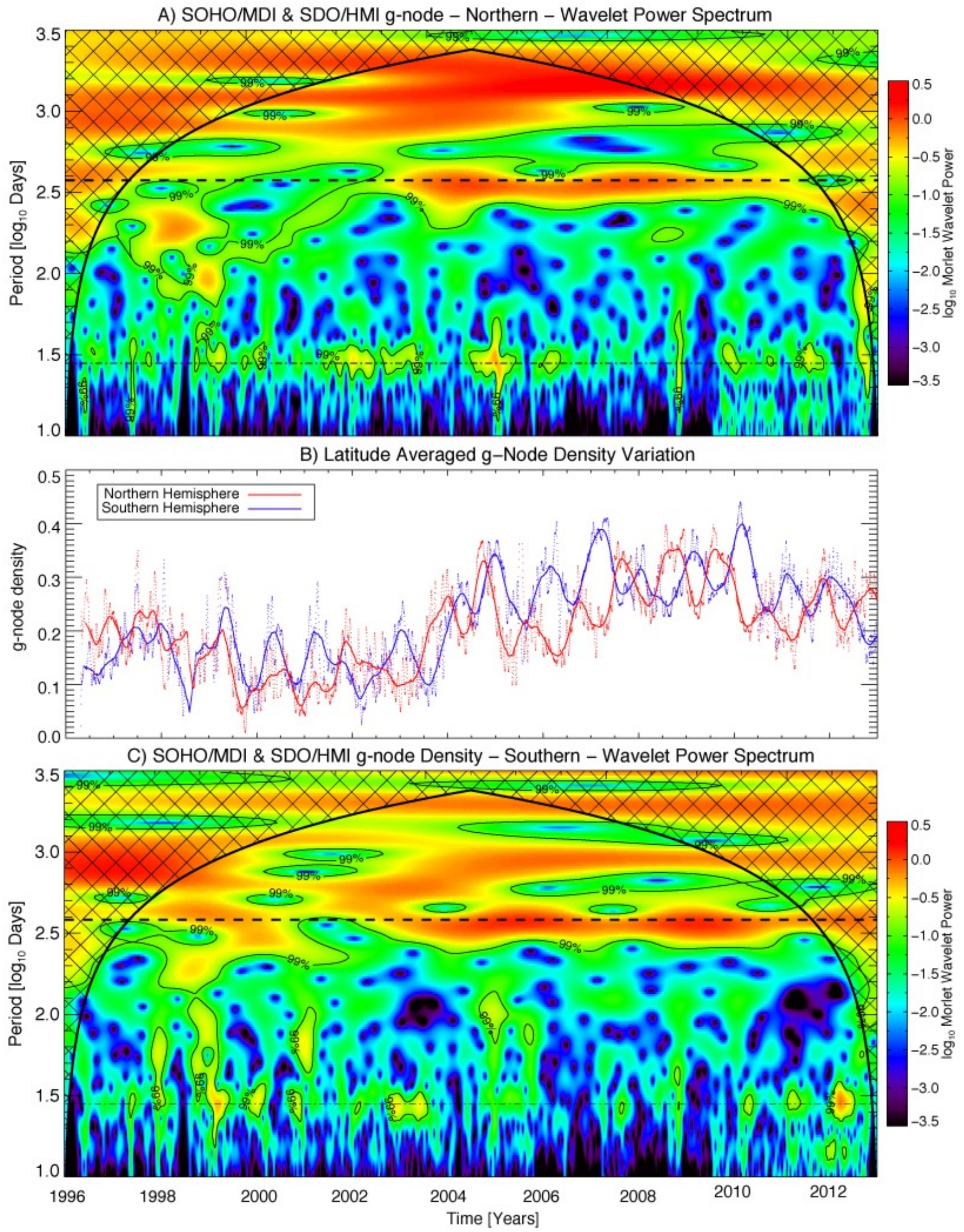

Supplementary Figure 1: The Morlet wavelet power spectra of the hemispheric g-Node density timeseries (Fig. 7A). Panels A and C show the power spectrum of the northern and southern hemispheric g-Node density timeseries respectively while panel B shows the individual timeseries. Panels A and C show the "Cone Of Influence" for the timeseries (the region of the plot that is not cross-hatched) and highlight the periods with a 99% confidence black using closed black contours. For reference we illustrate a period of 365 days as a thick dashed horizontal black line and 28 days as a thin dot-dashed horizontal black line.

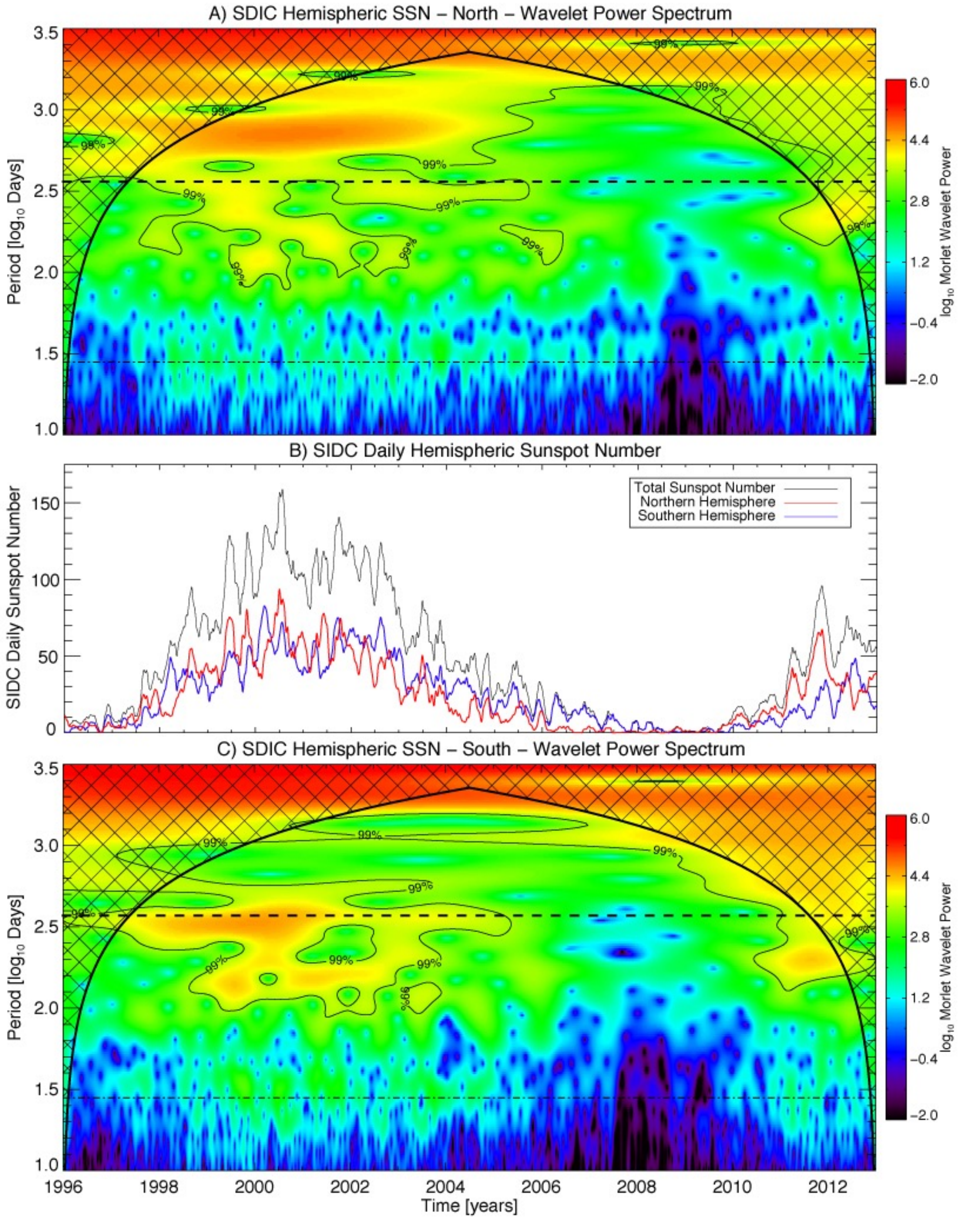

Supplementary Figure 2: The Morlet wavelet power spectra of the daily SIDC hemispheric SSN timeseries (Fig. 2B). Panels A and C show the power spectrum of the northern and southern timeseries respectively while panel B shows the individual timeseries in addition to the total sunspot number. Panels A and C show the "Cone Of Influence" for the timeseries (the region of the pilot that is not cross-hatched) and highlight the periods with a 99% confidence black using closed black contours. For reference we illustrate a period of 365 days as a thick dashed horizontal black line and 28 days as a thin dot-dashed horizontal black line.

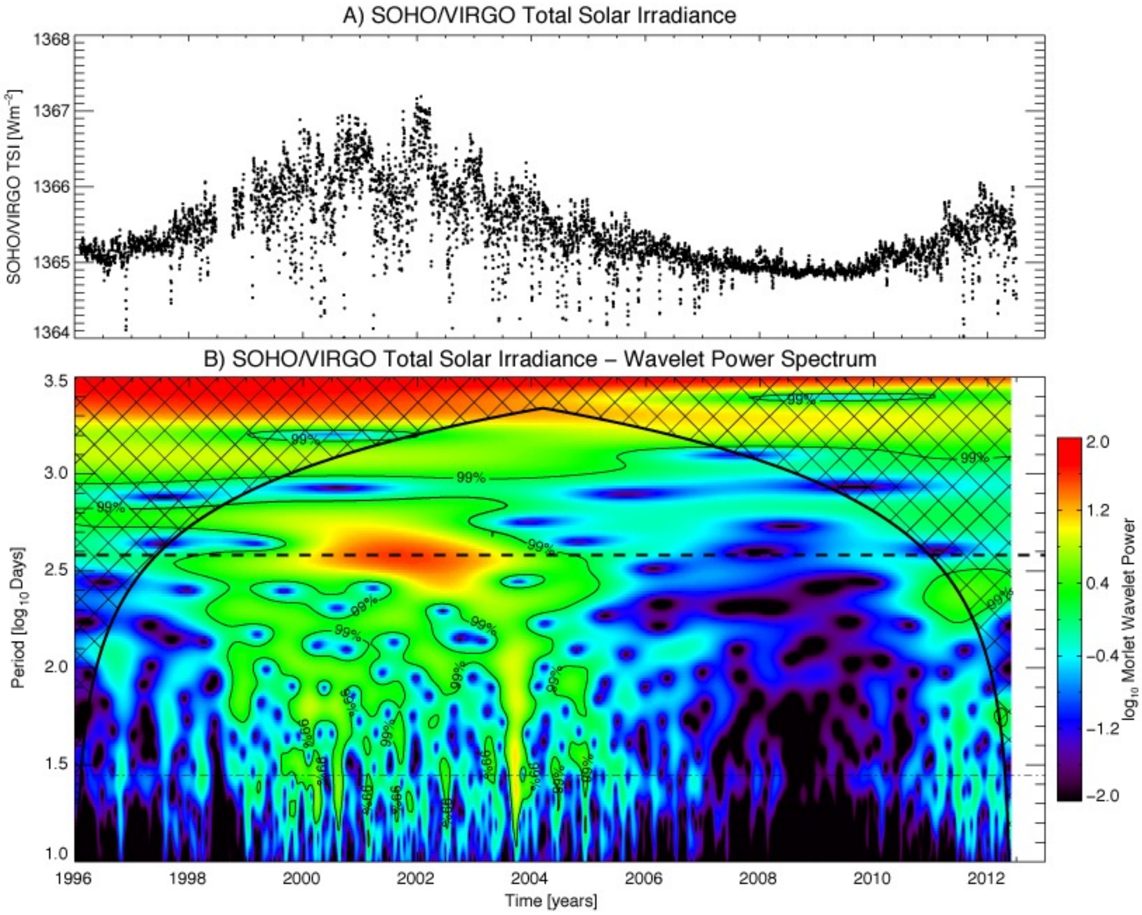

Supplementary Figure 3: The Morlet wavelet power spectra of the *SOHO*/ VIRGO TSI timeseries (Fig. 4B). Panel A shows the TSI timeseries while panel B shows the Wavelet power spectrum of the TSI timeseries. Panel B shows the "Cone Of Influence" for the timeseries (the region of the plot that is not cross-hatched) and highlight the periods with a 99% confidence black using closed black contours. For reference we illustrate a period of 365 days as a thick dashed horizontal black line and 28 days as a thin dot-dashed horizontal black line.
